# Femoral Head Fracture Without Associated Hip Dislocation

**DOI:** 10.1016/j.artd.2021.02.007

**Published:** 2021-03-11

**Authors:** Ashraf Shaikh, Mohan Desai, Radhakrishna Kantanavar, Kunal Shah

**Affiliations:** Department of Orthopaedics, Seth G.S. Medical College and K.E.M. Hospital, Parel, Mumbai, India

**Keywords:** Femoral head fracture, Indentation fracture, Head fracture without hip dislocation, Total hip arthroplasty, Rare injuries of hip

## Abstract

We present a rare case of femoral head indentation fracture without associated hip dislocation in an elderly female secondary to a low-energy trauma. She was managed with primary total hip arthroplasty and achieved an excellent functional outcome. We have proposed the mechanism of injury and discussed shortcomings in the present classification systems. A review of literature of such cases is presented along with the rationale for our management decision and the various other treatment modalities available for the surgeon.

## Introduction

First described by Birkett in 1869 in a postmortem dissection, femoral head fractures represent a rare injury which is almost always associated with hip dislocation [1,2]. It typically results from high-energy trauma due to motor vehicle accidents and are usually associated with acetabular or femoral neck fractures. The severity and pattern of the fracture are determined by the position of the hip (flexion, abduction or adduction, and rotations), direction, and the amount of the force during the traumatic event.

Isolated femoral head fractures after low-energy trauma are even rarer injuries. An extensive literature search has shown only 2 such reported cases [3,4]. The controversies regarding the ideal treatment of these fractures and the absence of a standard classification system with prognostic significance have resulted in the lack of guidelines for their optimal management.

We report one such case and review the typical pathophysiology and the proposed mechanism of this rare injury along with the management options.

A written informed consent was taken from the patient to publish this case report.

### Case history

A 70-year-old, previously healthy woman presented to the outpatient department in a wheelchair with complaints of pain in the left hip after an accidental fall at home 8 days back. She was nonambulatory and unable to bear weight since then. On clinical examination, there was significant tenderness at the hip joint. Although active range of motion was painful and restricted, passive range was full compared to the normal side.

There was no limb length discrepancy, and she had no distal neurovascular deficit. The patient did not have constitutional symptoms such as fever or weight loss nor was there any history of prolonged drug therapy, a connective tissue disorder, or ligamentous laxity on physical examination.

**Figure 1:**
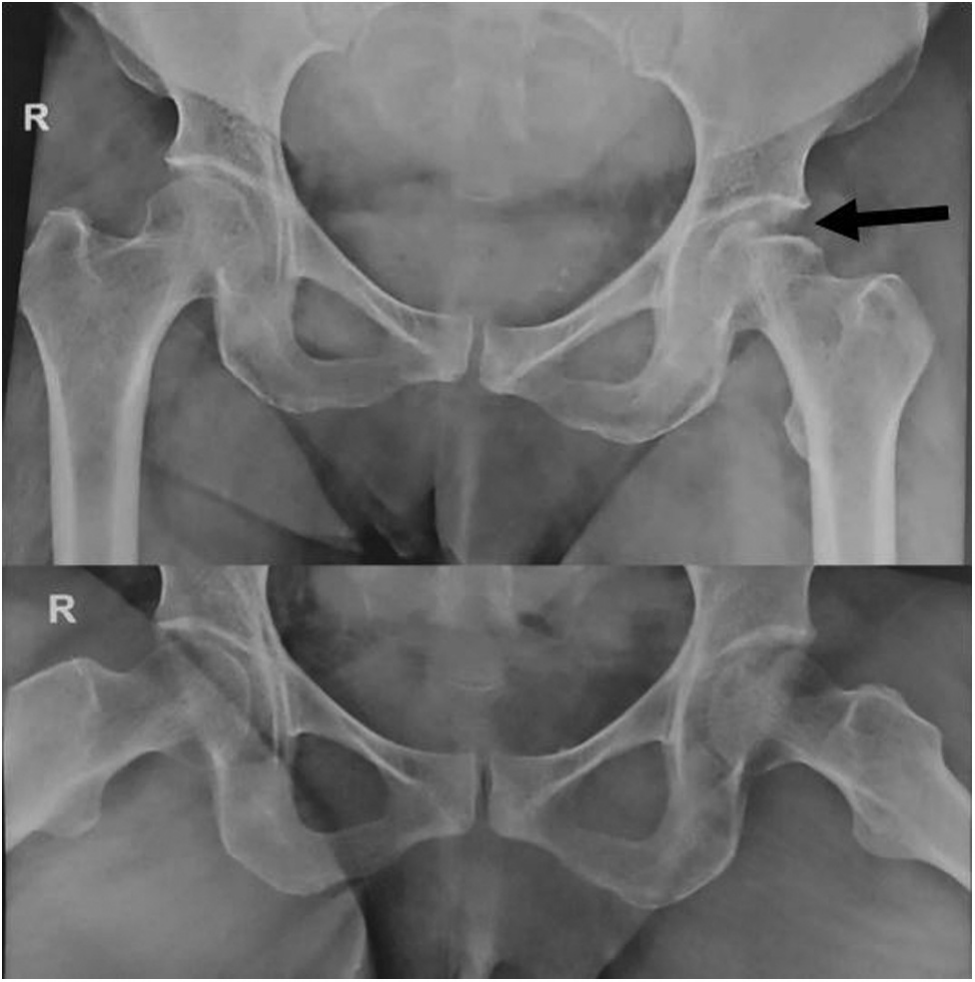
Indentation fracture seen on the superolateral surface of the femur head (arrow).

Radiological examination surprisingly demonstrated a femoral head indentation at the superolateral aspect without hip dislocation. There were no associated acetabulum or femoral neck fractures (Fig. 1). The femoral head showed no sclerotic or cystic changes, and there were no signs of secondary osteoarthritis as well. The femoral bone quality was good (Dorr Type A) with no signs of osteoporosis, thus ruling out a subchondral insufficiency fracture.

**Figure 2:**
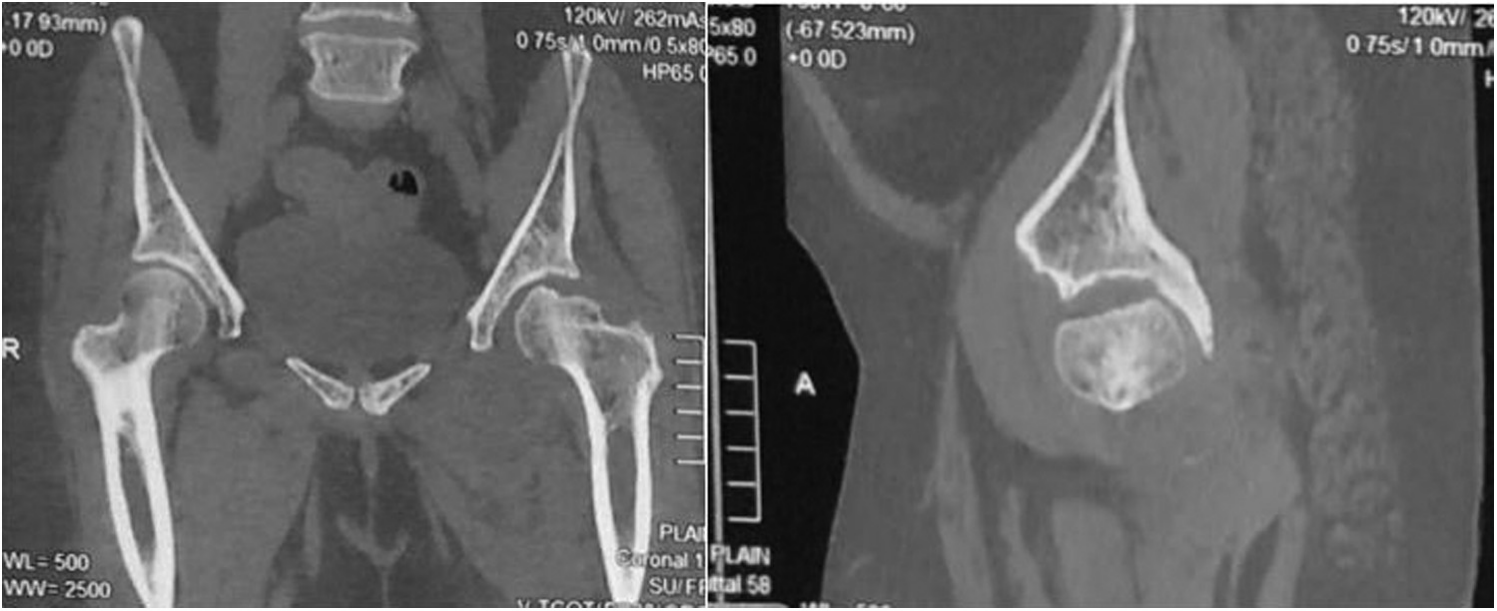
Coronal and sagittal sections of computed tomography scan confirming radiograph findings.

**Figure 3:**
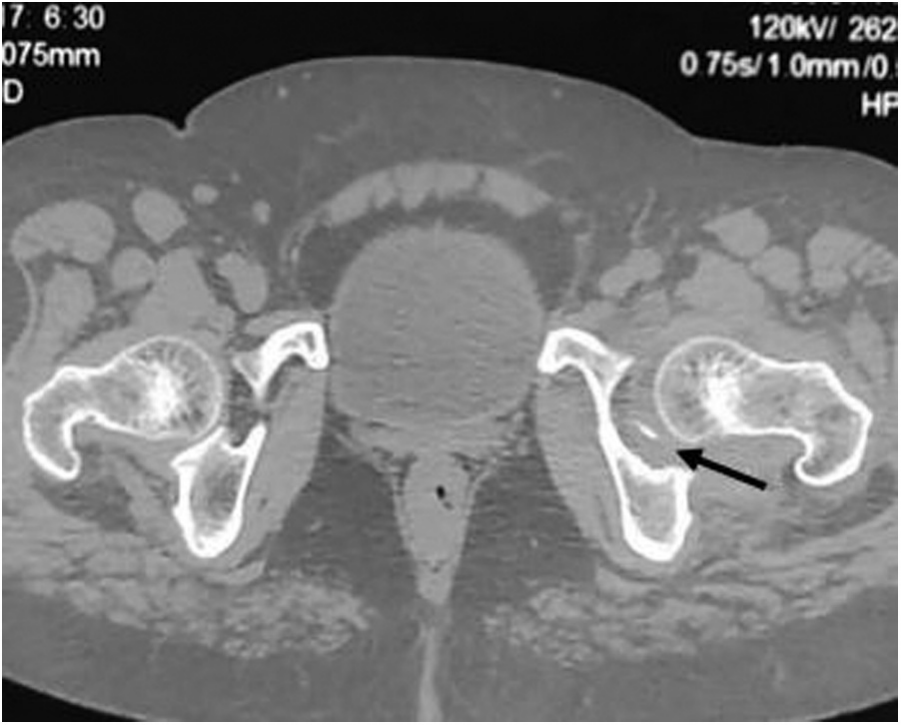
A small fractured fragment seen in the axial section (arrow).

Computed tomography scan confirmed the radiographic findings (Fig. 2) revealing a wedge-shaped cortical depression at the superolateral aspect of the femoral head. A small intra-articular osteochondral fragment was seen in the axial sections (Fig. 3).

**Figure 4:**
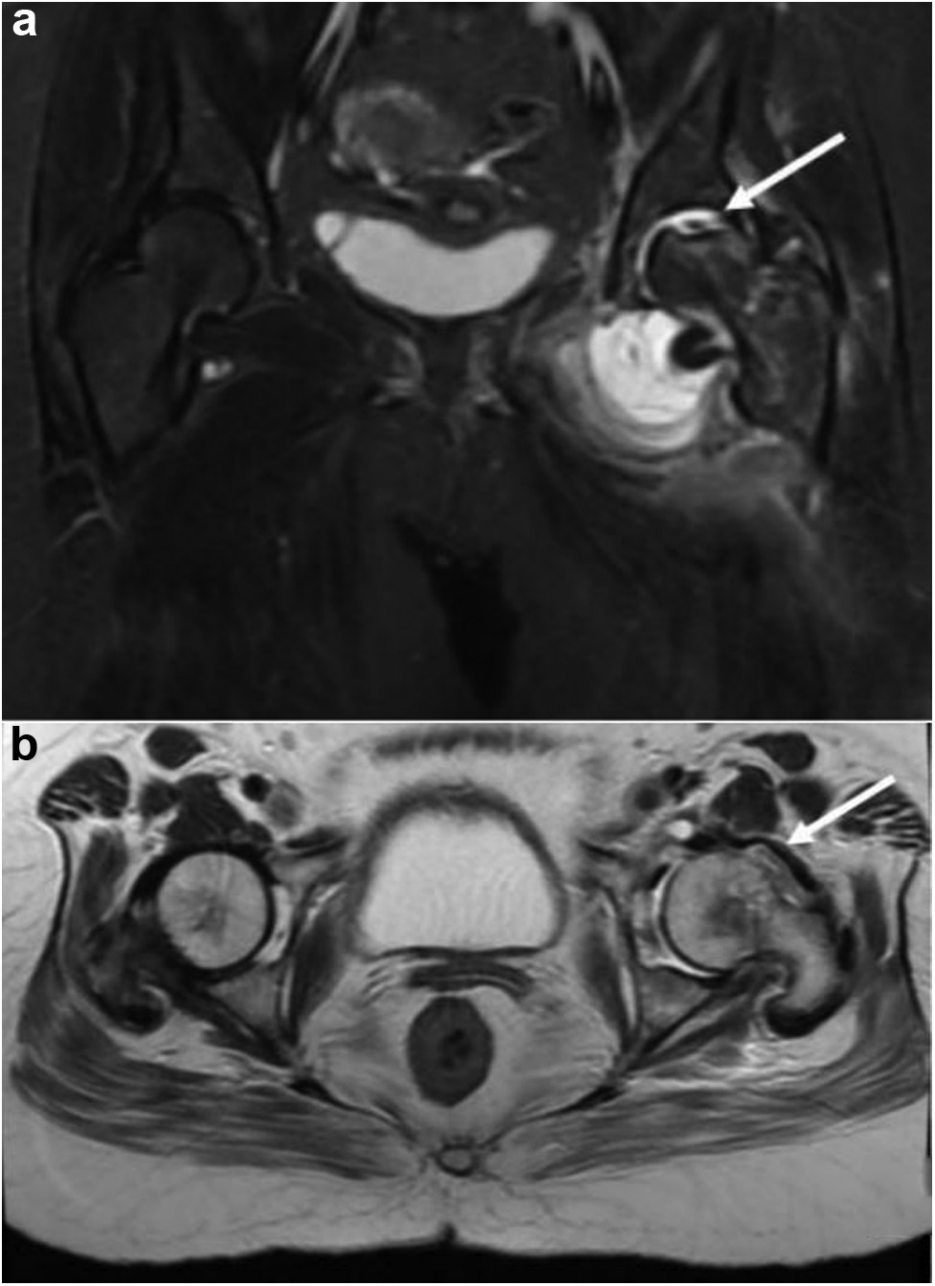
(a) Coronal STIR MR and (b) axial T2 MR: depressed subchondral posterosuperior femoral head fracture with surrounding marrow edema in head, subcapital region, neck, and intertrochanteric region (arrow). MR, magnetic resonance.

**Figure 5:**
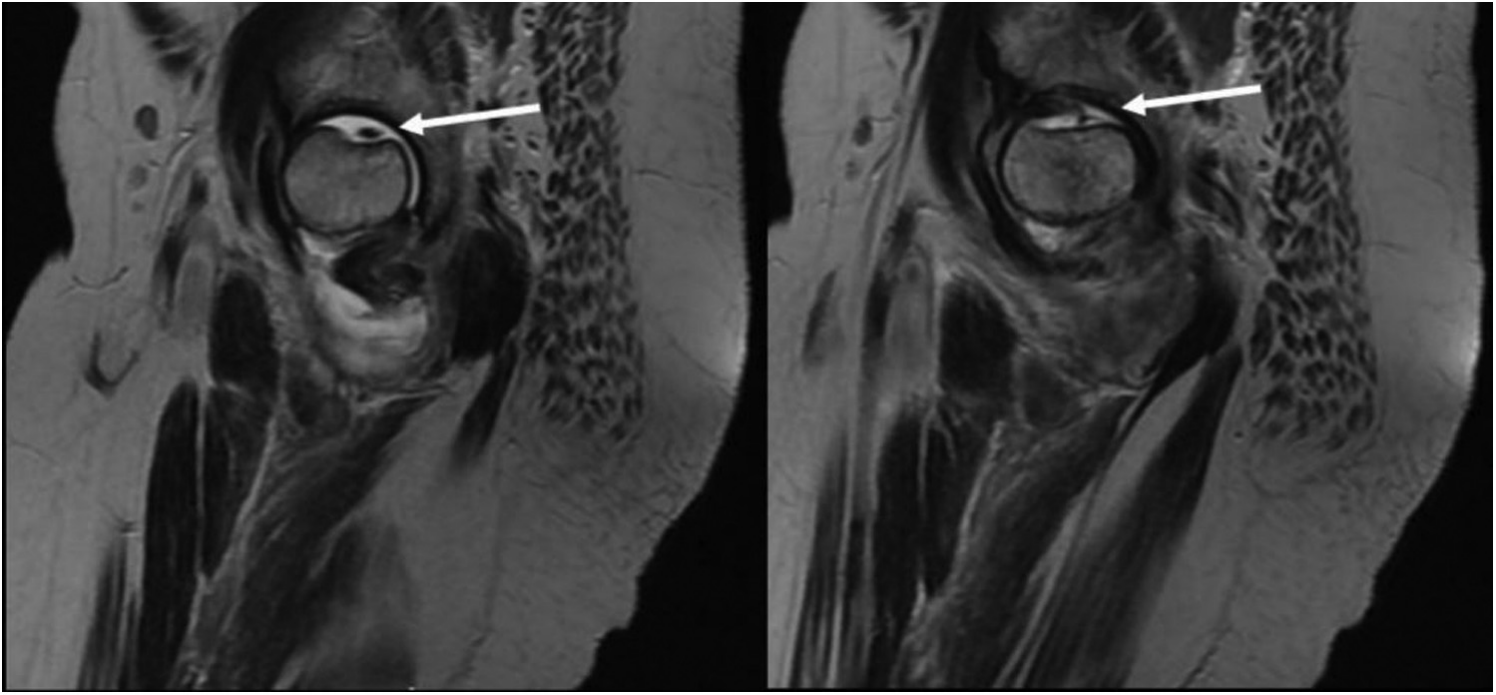
Sagittal T2 MR: Two consecutive cuts demonstrating the fragment as well as the indentation in the femur head (arrow). MR, magnetic resonance.

Magnetic resonance imaging (MRI) revealed a depressed subchondral posterosuperior femoral head fracture with surrounding marrow edema in head, subcapital region, neck, and intertrochanteric region (Figure 4, Figure 5). Short tau inversion recovery (STIR) hyperintense signal was seen in the medial acetabular cavity and left superior pubic ramus. A moderate left hip effusion with mild synovial thickening was seen. Diffuse subcutaneous and intermuscular edema was noted around the hip joint. The ligamentum teres as well as the capsulo-labral complex were intact all around the hip joint. The MRI reporting by the senior radiologist suggested posttraumatic or infective etiology and advised clinical correlation.

All routine hematological investigations were within normal limits including inflammatory markers such as erythrocyte sedimentation rate and the C-reactive protein.

**Figure 6:**
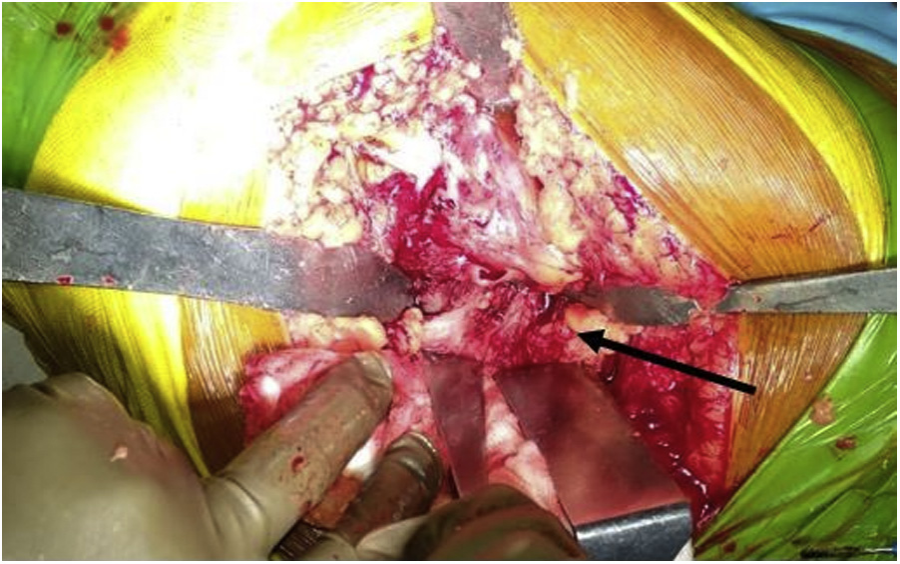
Intact short external rotators (arrow).

**Figure 7:**
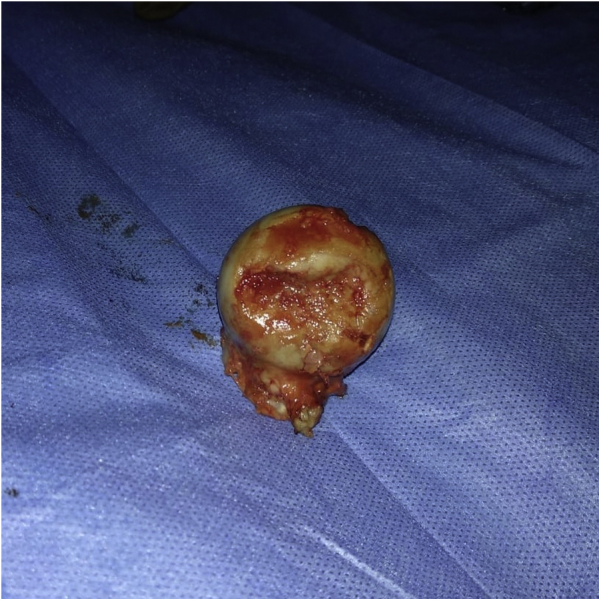
Indentation fracture on the superolateral surface of the femur head.

**Figure 8:**
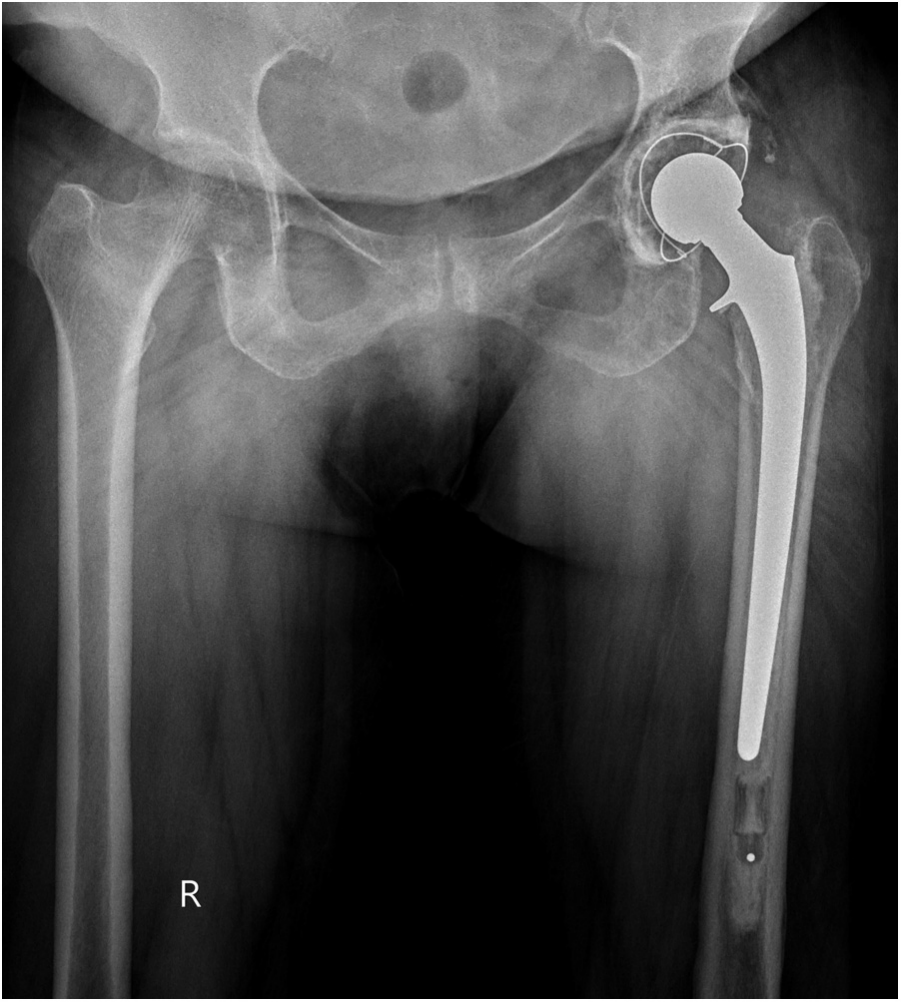
A cemented total hip arthroplasty performed with a collared stem.

A total hip arthroplasty (THA) was planned. On dynamic stress examination under anesthesia, the patient had full range of motion without hip instability. The authors prefer the modified Gibson’s approach for their primary hip arthroplasties. Intraoperatively, the short external rotators were found to be intact (Fig. 6). On lifting them, the posterior capsulolabral complex was completely intact as well. Apart from this, no rent was observed in the anterior as well as inferior capsule. We were unable to find the small, fractured fragment inside the hip joint which was visible in the axial sections of the computed tomography scan. The findings of an indentation fracture were confirmed on delivering out the otherwise normal femoral head (Fig. 7). A cemented THA was performed with a collared stem (Fig. 8). Intraoperatively sent tissue cultures were negative.

The patient was mobilized full weight-bearing with support from the next day after surgery. There was a dramatic relief in her pain. At 1-year follow-up, the functional outcome of patient was excellent. The Harris Hip Score at 1 year was 79, which was a significant improvement from the preoperative score of 28.

## Discussion

The hip is an intrinsically stable joint, demanding a substantial amount of force to dislocate. Therefore, pure hip dislocations or dislocations with femoral head fractures are largely a product of high-energy trauma and are often accompanied with associated injuries. The incidence of femoral head fractures in cases of hip dislocations is around 5% to 15% [1]. However, femoral head fractures almost always occur in association with hip dislocation [1,2].

A posteriorly directed force to the knee with the hip flexed has been described as the mechanism of posterior hip fracture-dislocations [1]. Femoral head impaction against the posterior acetabular wall results in its fracture and dislocation of the remaining part posteriorly.

The mechanism of injury of anterior hip fracture-dislocations stems from a combination of flexion, abduction, and external rotation which leads to the inferior obturator dislocation. In contrast to this, abduction and external rotation along with extension of the hip results in the less frequent superior pubic dislocation.

**Figure 9:**
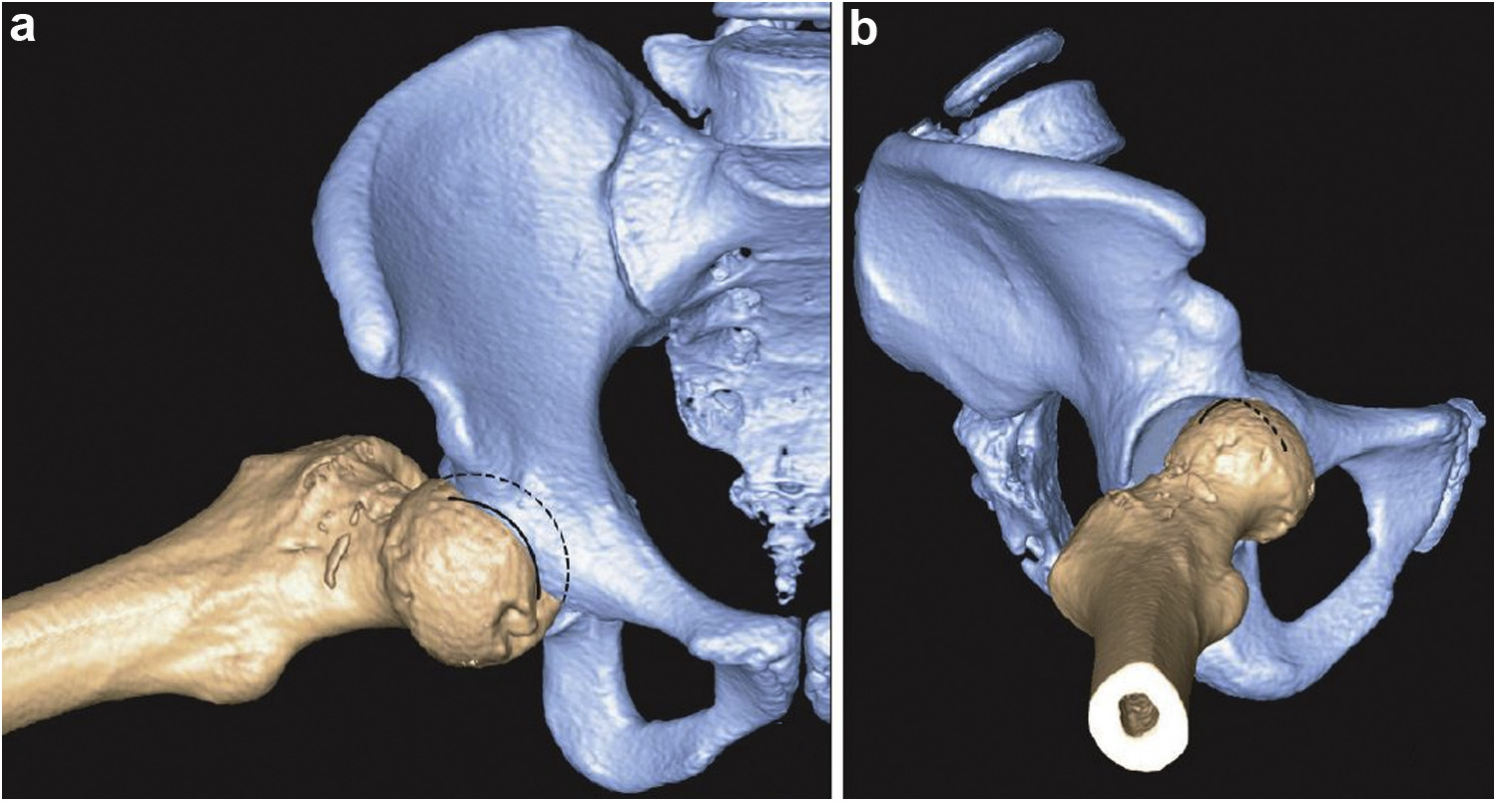
Computed tomography 3D reconstruction was used to simulate the proposed mechanism of fracture in this patient. (a) Fracture line of the patient is represented by the solid line whereas the dotted line represents the femoral head outline. (b) Femoral head fracture line seen from another view. This image has been taken from the report by Yoon et al. [4] with permission.

Our patient sustained a fall at home while getting up from the bed with her affected hip in abduction, external rotation, and flexion. Therefore, the proposed mechanism of injury is an anterior hip subluxation with head indentation fracture which got spontaneously relocated before she presented to us (Fig. 9).

In 1957, Pipkin [2] classified fractures of femoral head based on his observation of 24 patients, and even today, his classification is most commonly used for these fractures. Isolated femoral head fractures without hip dislocation are not included in his classification. Another detailed classification of femoral head fracture was given by Brumback et al. [5] in 1986. This classification system commented on the stability of the hip and guided the line of management; however, it failed to incorporate isolated fractures of femoral head.

In 2013, Aggarwal et al. [6] proposed a modification of the Pipkin classification wherein he divided femoral head fractures into type A and B. Type A fractures were not associated with hip dislocations and were further subclassified in the same way as in the Pipkin classification.

The AO classification does have room for isolated femoral head fractures; however, neither does it reflect the mechanism of injury nor does it guide the management, thus reducing its clinical utility.

After performing a thorough review of literature, we found only 8 case reports of femoral head fractures without hip dislocations. Out of the 8, only 4 were isolated femoral head fractures (without associated acetabulum or neck fractures), and out of these 4, only 2 occurred in a setting of low-energy trauma (Mody et al. and Yoon et al. [3,4]; Table 1)

**Table 1:** Review of literature of the published reports of femur head fractures without hip dislocations.

Sr. No.	Author	Age/Sex	Severity of trauma	Diagnosis	Management
1	Van der Werken and Blankensteijn (1987) [7]	25/M	High energy	Right acetabulum and left femoral head fracture	Conservative
2	Mody and Wainwright (1993) [3]	1.57/F 2. 53/F	1. Low energy 2. Low energy	1. Superolateral impacted femoral head fracture 2. Right inferomedial femoral head fracture	1. Total hip arthroplasty 2. ORIF
3	Fabre et al. (2003) [8]	26/M	High energy	Comminuted femoral head and neck fracture	Bipolar hip hemiarthroplasty
4	Matsuda (2009) [9]	19/M	High energy	Femoral head osteochondral fracture	Arthroscopic reduction and internal fixation
5	Yoon et al. (2011) [4]	21/M	Low energy	Superomedial femoral head fracture	Conservative
6	Aggarwal et al. (2013) [6]	36/M	High energy	Neglected (1 year old) comminuted femoral head fracture	Total hip arthroplasty
7	Kapil Pawar and Kandhari (2016) [10]	48/M	High energy	Femoral head and neck fracture	Uncemented total hip arthroplasty
8	Lee et al. (2019) [11]	33/M	Low energy	Femoral head and posterior acetabulum fracture	Conservative

F, female; M, male; ORIF, open reduction and internal fixation.

**Figure 10:**
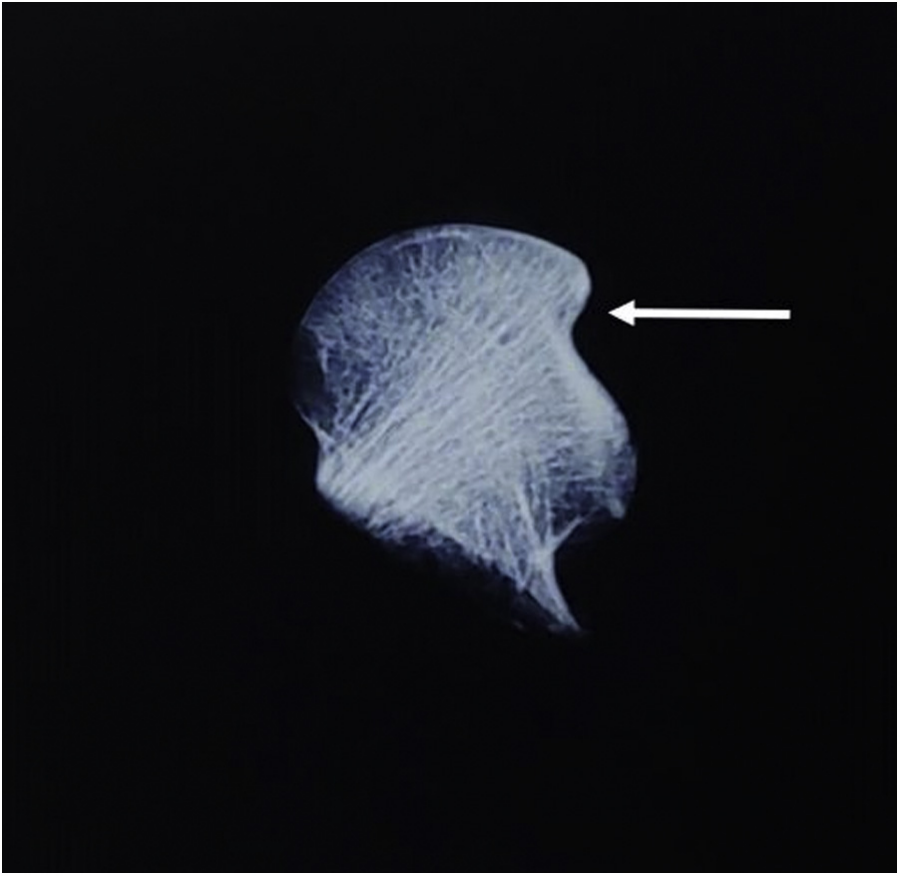
Radiograph of the excised femur head without any signs of osteonecrosis. Indentation is seen on the superolateral aspect (arrow).

The authors ruled out the diagnosis of osteonecrosis of hip as it typically occurs in the third-fourth decade of life, whereas the patient was 70 years old. There was no history of associated risk factors such as alcohol abuse, steroids, presence of systemic lupus erythematosus, or other connective tissue disorders. Moreover, the patient did not have any prodromal symptoms suggestive of osteonecrosis (ON) before the fall. The MRI failed to show any classical signs of ON (the double density sign). Intraoperatively, on examining the delivered-out femoral head, we did not find signs of osteonecrosis or arthritis. Radiological examination of the excised femoral head did not show sclerosis, cysts, or any other signs of ON (Fig. 10).

There are no universal guidelines for management of isolated femoral head fractures; however factors such as age of the patient, size, and location of the fragment play a huge role in treatment decisions.

The patient should be managed conservatively if the fragment is small and is not in the weight-bearing area (Pipkin I) [12]. Alternatively, the fragment can be excised or debrided without any fixation [1].

In young patients, an open reduction and internal fixation can be done when the fragment is large and involves the weight-bearing region (Pipkin II) [13]. Subarticular fixation is a must, thus necessitating the use of headless compression screws, countersinking of screws, or suture anchor repair [14]. The indentation fracture may need to be elevated and bone loss tackled with bone grafting using a window made in the non–weight-bearing portion of the femur head, a technique comparable to the “trapdoor” procedure carried out for osteonecrosis of hip [13].

The surgical approach for the fracture fixation is also controversial. The Kocher-Langenbeck approach has a higher rate of osteonecrosis and comes with difficulty in visualizing the fracture fragment [12]. Low incidence of osteonecrosis, easier reduction, and better visualization have been achieved with the Smith-Peterson approach; however, it has shown a higher rate of heterotopic ossification [14].

Our patient was a 70-year-old female having severe pain in her affected hip and was not able to bear weight. Before the injury, she was active and carried out activities of daily living independently. Fracture fixation in this patient would call for prolonged immobilization, not to mention the high probability of secondary osteoarthritis, subchondral collapse, failure of fixation, and osteonecrosis [15]. A THA later would lead to other difficulties such as the need for highly experienced surgical expertise and a financial and emotional burden to the patient. Therefore, we decided to go ahead with a primary THA with the benefits of a better clinical outcome, quick functional rehabilitation, and cost-effectiveness.

We performed a cemented fixation as it has a better overall long-term survivorship and a lower rate of complications than cementless fixation in primary THA in the elderly population [16,17].

Relatively few studies have been published on this type of injury because of its rarity and that explains the little evidence on it. Further research on mechanism of this rare injury, classification system which dictates the management plans, and validated measurements of outcomes will guide the surgeons to practice evidence-based orthopedics.

## Summary

Isolated femoral head fracture is a very rare injury to occur, and therefore, surgeons must rule out associated injuries. When truly isolated, it is best managed with THA in an elderly patient.

## Conflict of interests

The authors declare that they have no known competing financial interests or personal relationships that could have appeared to influence the work reported in this article.

## Appendix A Supplementary data

Conflict of Interest Statement for Shaikh

Conflict of Interest Statement for Shah

Conflict of Interest Statement for Desai

Conflict of Interest Statement for Kantanavar

## References

[1] Epstein H.C., Wiss D.A., Cozen L. (1985). Posterior fracture dislocation of the hip with fractures of the femoral head. Clin Orthop.

[2] Pipkin G. (1957). Treatment of grade IV fracture-dislocation of the hip. J Bone Joint Surg Am.

[3] Mody B.S., Wainwright A.M. (1996). Fracture of the femoral head without associated hip dislocation following low-energy trauma: a report of two cases. Arch Orthop Trauma Surg.

[4] Yoon P.W., Jeong H.S., Yoo J.J., Koo K.H., Yoon K.S., Kim H.J. (2011). Femoral head fracture without dislocation by low-energy trauma in a young adult. Clin Orthop Surg.

[5] Brumback R.J., Kenzora J.E., Levitt L.E. (1986). Fractures of the femoral head. Proceedings of the hip society.

[6] Aggarwal A.K., Soni A., Singh D. (2013). Femoral head fracture without hip dislocation. Chin J Traumatol.

[7] Van der Werken C., Blankensteijn J.D. (1987). Fracture of the femoral head without dislocation: a case report. Acta Orthop Scand.

[8] Fabre A., Bures C., Levadoux M., Leguilloux P., Rigal S. (2003). A comminuted femoral head fracture without hip dislocation. Eur J Orthop Surg Traumatol.

[9] Matsuda D.K. (2009). A rare fracture, an even rarer treatment: the arthroscopic reduction and internal fixation of an isolated femoral head fracture. Arthroscopy.

[10] Pawar K., Kandhari V.K. (2016). A rare medley: concurrent ipsilateral femur head and neck fracture without hip dislocation. J Surg Case Rep.

[11] Lee Y.-K., Min J.J., Koo K.-H. (2019). Pipkin type IV femoral head fracture without dislocation. Ann Surg Case Rep.

[12] Swiontkowski M.F., Thorpe M., Seiler J.G., Hansen S.T. (1992). Operative management of displaced femoral head fractures: case-matched comparison of anterior versus posterior approaches for Pipkin I and Pipkin II fractures. J Orthop Trauma.

[13] Giordano V., Giordano M., Glória R.C. (2019). General principles for treatment of femoral head fractures. J Clin Orthop Trauma.

[14] Henle P., Kloen P., Siebenrock K.A. (2007). Femoral head injuries: which treatment strategy can be recommended?. Injury.

[15] Giannoudis P.V., Kontakis G., Christoforakis Z., Akula M., Tosounidis T., Koutras C. (2009). Management, complications and clinical results of femoral head fractures. Injury.

[16] Zhang C., Yan C.H., Zhang W. (2017). Cemented or cementless fixation for primary hip arthroplasty–evidence from the International Joint Replacement Registries. Ann Joint.

[17] Blankstein M., Lentine B., Nelms N.J. (2020). The use of cement in hip arthroplasty: a contemporary perspective. J Am Acad Orthop Surg.

