# Supplementary material for: Femoral Head Fracture Without Associated Hip Dislocation
Source: Arthroplast Today. 2021 Mar 11;8:145–9. doi: 10.1016/j.artd.2021.02.007 (PMC7966925; doi:10.1016/j.artd.2021.02.007)
Supplement: Conflict of Interest Statement for Shaikh [file mmc1.pdf]

# CONFLICT OF INTEREST STATEMENT

## *American Association of Hip and Knee Surgeons*

(Adopted from the American Academy of Orthopaedic Surgeons disclosure statement)

Manuscript Title **Femoral head fracture without associated hip dislocation – A case report and review of literature**

1. Royalties from a company or supplier **NONE**

2. Speakers bureau/paid presentations for a company or supplier **NONE**

3A. Paid employee for a company or supplier **NONE**

3B. Paid consultant for a company or supplier **NONE**

3C. Unpaid consultants for a company or supplier **NONE**

4. Stock or stock options in a company or supplier **NONE**

5. Research support from a company or supplier as a Principal Investigator **NONE**

6. Other financial or material support from a company or supplier **NONE**

7. Royalties, financial or material support from publishers **NONE**

8. Medical/Orthopaedic publications editorial/governing board **NONE**

9. Board member/committee appointments for a society **NONE**

**Each author must sign AND print or type his/her name, date and submit a separate form**

In addition, one BLINDED Conflict of Interest form (no author names used) should be submitted per manuscript with all author disclosures.

| Author Name | Author Signature | Date 21.1.2020 |
|---|---|---|
| Dr. Ashraf Shaikh | [signature] | |
