# Supplementary material for: Femoral Head Fracture Without Associated Hip Dislocation
Source: Arthroplast Today. 2021 Mar 11;8:145–9. doi: 10.1016/j.artd.2021.02.007 (PMC7966925; doi:10.1016/j.artd.2021.02.007)
Supplement: Conflict of Interest Statement for Shah [file mmc2.pdf]

CONFLICT OF INTEREST STATEMENT

American Association of Hip and Knee Surgeons

(Adopted from the American Academy of Orthopaedic Surgeons disclosure statement)

Manuscript Title **Femoral head fracture without associated hip dislocation – A case report and review of literature**

1. Royalties from a company or supplier **NONE**

2. Speakers bureau/paid presentations for a company or supplier **NONE**

3A. Paid employee for a company or supplier **NONE**

3B. Paid consultant for a company or supplier **NONE**

3C. Unpaid consultants for a company or supplier **NONE**

4. Stock or stock options in a company or supplier **NONE**

5. Research support from a company or supplier as a Principal Investigator **NONE**

6. Other financial or material support from a company or supplier **NONE**

7. Royalties, financial or material support from publishers **NONE**

8. Medical/Orthopaedic publications editorial/governing board **NONE**

9. Board member/committee appointments for a society **NONE**

Each author must sign AND print or type his/her name, date and submit a separate form

In addition, one BLINDED Conflict of Interest form (no author names used) should be submitted per manuscript with all author disclosures.

Author Name	Author Signature	Date
Dr. Kunal Shah	Kunal Shah	21.1.2020
