# Supplementary material for: Femoral Head Fracture Without Associated Hip Dislocation
Source: Arthroplast Today. 2021 Mar 11;8:145–9. doi: 10.1016/j.artd.2021.02.007 (PMC7966925; doi:10.1016/j.artd.2021.02.007)
Supplement: Conflict of Interest Statement for Desai [file mmc3.pdf]

CONFLICT OF INTEREST STATEMENT

American Association of Hip and Knee Surgeons

(Adopted from the American Academy of Orthopaedic Surgeons disclosure statement)

[illegible]anuscript Title **Femoral head fracture without associated hip dislocation – A case report and review of literature**

[illegible] Royalties from a company or supplier **NONE**

[illegible] Speakers bureau/paid presentations for a company or supplier **NONE**

[illegible]A. Paid employee for a company or supplier **NONE**

[illegible]B. Paid consultant for a company or supplier **NONE**

[illegible]C. Unpaid consultants for a company or supplier **NONE**

[illegible] Stock or stock options in a company or supplier **NONE**

[illegible] Research support from a company or supplier as a Principal Investigator **NONE**

[illegible] Other financial or material support from a company or supplier **NONE**

[illegible] Royalties, financial or material support from publishers **NONE**

[illegible] Medical/Orthopaedic publications editorial/governing board **NONE**

[illegible] Board member/committee appointments for a society **NONE**

[illegible]ach author must sign AND print or type his/her name, date and submit a separate form

[illegible]n addition, one BLINDED Conflict of Interest form (no author names used) should be submitted per manuscript with all [illegible]uthor disclosures.

[illegible]uthor Name	Author Signature	Date 21.1.2020
[illegible]r. Mohan Desai	M. M. Desai	
